# Narrow-linewidth photonic wirebonded silicon nitride external cavity tunable laser

**DOI:** 10.1038/s41598-026-50776-9

**Published:** 2026-05-01

**Authors:** David A. S. Heim, Gar-Wing Truong, Debapam Bose, Eduardo Diaz, Juan Ramirez, Jes Sherman, Gordon Morrison, Daniel J. Blumenthal

**Affiliations:** 1 Department of Electrical and Computer Engineering, University of California Santa Barbara, Santa Barbara, CA 93106 USA; 2 Freedom Photonics, 41 Aero Camino, Santa Barbara, CA 93117 USA

**Keywords:** Engineering, Optics and photonics, Physics

## Abstract

Ultra-low linewidth widely tunable lasers capable of emission by design from the visible to shortwave infrared are important building blocks for a range of precision applications including quantum sensing and computing, timekeeping, metrology, optical clocks, and fiber sensing. Importantly, integration of precision tunable lasers in a CMOS foundry compatible platform that can support higher level integration with other components, such as low loss silicon nitride (Si_3_N_4_), is an important step towards full system on chip solutions. Integration of the III-V gain material with the Si_3_N_4_ tunable cavity is a critical step towards this goal and must be achieved through a low-cost, manufacturable, and reliable process. However, this co-integration has remained challenging due to tight alignment tolerances and mode mismatches between the semiconductor and silicon nitride waveguides. 3D-printed photonic wire bonding (PWB) offers a robust approach to hybrid integration due to the relaxation of waveguide alignment tolerances and the inherent low-loss mode matching. In this work, we demonstrate a narrow linewidth PWB-integrated Si_3_N_4_ external cavity tunable laser (ECTL) with a 3.75—7.77 Hz fundamental linewidth measured across a 60 nm tuning range and a 1.27 kHz integral linewidth: a reduction of nearly three orders of magnitude in fundamental linewidth compared with previously reported PWB-integrated ECTLs in Si_3_N_4_. The PWB process has the potential to realize reliable and manufacturable tunable lasers on-chip with the performance of table-top fiber lasers. These results establish photonic wirebonding as a viable integration pathway for precision photonic systems, enabling portable, scalable, and cost-effective solutions for quantum, low-noise microwave, and sensing applications.

**Supplementary Information:**

The online version contains supplementary material available at 10.1038/s41598-026-50776-9.

## Introduction

Hybrid integration of microfabricated photonic devices manufactured on different material platforms holds the promise of realizing the best aspects of different materials into one co-packaged assembly. The ultra-low loss silicon nitride (Si_3_N_4_) integration platform^1^ can realize precision high-performance widely tunable lasers on-chip that rival the performance of table-top fiber lasers^2^. Yet, integration of the active gain material with the tunable laser cavity in this platform, while maintaining low insertion loss and narrow linewidth performance, has remained challenging. Monolithic laser designs, that provide direct integration of the gain material and wide tunability^3^ can benefit from replacement of the extended cavity with Si_3_N_4_ to enable linewidth narrowing and frequency noise reduction. An emergent technique towards this goal is hybrid integration with photonic wire bonding (PWB), wherein printed polymer waveguides are used to connect the optical inputs and outputs between photonic devices. The PWB simultaneously functions as an optical interconnect and a mode converter, thereby enhancing integration flexibility. Other functionalities that have been demonstrated with PWBs include broadband polarization-dependent beam splitting^4^, n-port couplers for combining multiple outputs of a bar of semiconductor lasers^5^, and printed microlenses^6^. Here we demonstrate the integration of an InP-based reflective semiconductor optical amplifier (RSOA) with a low-loss Si_3_N_4_ external cavity tunable laser (ECTL) to realize a fully integrated laser with single-digit Hz fundamental linewidth (FLW) measured across a 60 nm tuning range, covering the entire C-band. Previous PWB-ECTLs in Si_3_N_4_ have been limited to, at best, 59 dB sidemode suppression ratio (SMSR) and just under 1 kHz FLW^7^, with a dual-gain TFLN implementation reaching 61 dB SMSR with 550 Hz FLW^8^ (see Supplementary Table S1). This work achieves record-low FLW of 3.75 Hz and record-high SMSR of 69.5 dB among PWB-based ECTLs, representing a significant advance in the applicability of PWB integration for precision technologies.

## Device design and fabrication

**Fig. 1:**
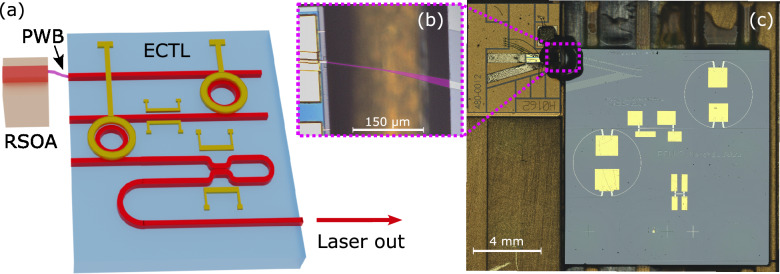
(**a**) Illustration of the hybrid-integrated external cavity tunable laser (ECTL) layout. The silicon nitride (Si_3_N_4_) external cavity consists of two thermo-optically tuned Vernier rings and a Sagnac loop mirror. A reflective SOA (RSOA) is optically connected to the Si_3_N_4_ chip via a photonic wirebond (PWB). (**b**) Microscope image of the photonic wirebond (false colored purple) that connects the RSOA to the Si_3_N_4_ photonic chip. (**c**) Microscope image of the PWB-integrated ECTL.

The PWB-integrated ECTL (PWB-ECTL) is based on an ECTL design reported in^2^ and is illustrated in Fig. 1(a), with a photograph of the PWB and hybrid-integrated device shown in Fig. 1(b) and (c), respectively. The laser cavity incorporates two thermo-optically tuned high-quality factor (Q) ring resonators in an add-drop Vernier configuration, a tunable phase section, a Sagnac loop mirror reflector, and an InP RSOA coupled via a photonic wire bond. The intracavity rings (intrinsic Q ≈ 3.5 × 10^6^) provide extended photon lifetimes for linewidth narrowing, while the Vernier effect enables broadband single mode operation across ~ 60 nm. Each ring–bus coupler is designed with a power coupling coefficient of κ^2^ ≈ 0.4, chosen to achieve over-coupling and reduce the lasing threshold. The Sagnac loop mirror supplies a broadband reflection, and the thermo-optic phase section enables fine longitudinal mode selection. The Si_3_N_4_ waveguides are 2.8 µm wide and taper out to 18 µm at the facet interfacing with the photonic wirebond. This facet geometry and its input angle (13.1°) were optimized for free-space edge coupling to the RSOA used in^2^ and were not modified for PWB compatibility. While this waveguide width is non-optimal for PWB – requiring aggressive tapers in the mode conversion section – it demonstrates the design flexibility afforded by the PWB integration approach to accommodate non-ideal geometries.

The photonic wirebond comprises three distinct sections: an input taper mode-matched to the RSOA, an output taper mode-matched to the ECTL chip, and a near-single-mode section that connects the two tapers. The anti-reflection (AR) coated facet of the 2-mm-long RSOA is angled at 10.5° from normal, and the PWB is curved to meet the ECTL chip facet at 12.6°, accounting for the refractive index difference between the polymer and the surrounding medium. Simulations yield a mode-matching loss of 0.6 dB at the PWB-Si_3_N_4_ interface and 0.7 dB at the PWB-RSOA interface, for a total ideal mode-matching loss of 1.3 dB, excluding propagation and bending losses. The experimentally achieved coupling loss is estimated to be closer to 7 dB, determined from direct loss measurements on a test assembly consisting of a similar RSOA and Si_3_N_4_ waveguide taper. This excess loss is attributed to positional uncertainty in locating the waveguide layer depth during PWB writing (see Supplementary Fig. S1). Additional experimental optimization of the bond placement to better account for imaging tolerances in determining the waveguide layer depth in both RSOA and ECTL is expected to reduce this loss; prior work has demonstrated PWB coupling losses as low as 1.7 dB^9^.

The separation between the RSOA and ECTL chip was set to approximately 300 µm by the mount geometry, defining the distance that is bridged by the PWB. Lateral placement of the RSOA and ECTL was controlled using encoded positioners and an optical microscope. Precise height matching and in-plane alignments are not critical due to the geometric flexibility of the PWB process. Although tighter limits apply to any particular bond, the photonic wirebonding tool (Vanguard Sonata 1000) has a writable volume of approximately 320 × 325 × 200 μm. A custom-machined aluminum mount measuring 17 mm × 31 mm was used to fix the RSOA, ECTL, and output polarization-maintaining (PM) fiber on a common carrier. The mount geometry was designed to compensate for the height mismatches between components, bringing the waveguide layers and fiber core into a common plane. The output fiber was actively aligned and secured to the mount at the position of maximum ECTL output coupling.

## Results

### Lasing characteristics

**Fig. 2:**
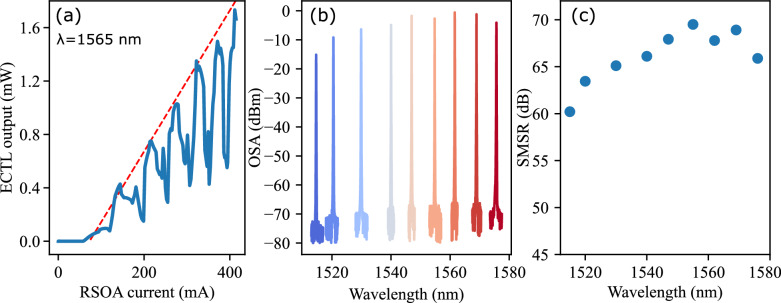
(**a**) Output power of the ECTL in packaged fiber as a function of the reflective SOA (RSOA) drive current (blue) indicating a lasing threshold current of ~ 73 mA. The phase and ring heaters were kept fixed across the current sweep; as a result, discontinuities appear due to longitudinal mode hopping. The red-dashed line indicates the expected linear above-threshold behavior, shown as an idealized guide to the eye. (**b**) Single mode operation of the ECTL measured on an optical spectrum analyzer (OSA) and tuned by adjusting the current applied to the Vernier ring heaters. (**c**) Measured side-mode suppression ratio (SMSR) of the external cavity tunable laser (ECTL) across the full 60 nm tuning range. The SMSR is limited by the noise floor of the OSA.

The performance of the PWB-integrated ECTL is summarized in Fig. 2. The laser exhibits a lasing threshold of 73 mA (Fig. 2(a)), measured at 1565 nm. The two intracavity rings with radii of 1998.36 and 2002.58 μm yield a Vernier free-spectral range (FSR) of 60 nm, corresponding to the wavelength separation between their overlapping resonances. Single longitudinal mode operation is measured across 1515–1576 nm (Fig. 2(b)). The operating wavelength is controlled by applying current to the thermo-optic heaters on the Vernier rings with a measured tuning efficiency of 50 nm/W. The Sagnac loop mirror reflectivity is determined by the wavelength-dependent power coupling coefficient κ^2^ of the directional coupler. Simulations of our waveguide geometry (thickness: 80 nm, width: 2.8 µm, gap: 2.5 µm, coupling length: ~ 575 µm) yield κ^2^ increasing from 0.7 to 0.9 across the ECTL operating range, and since Sagnac reflectivity is maximized when κ^2^ = 0.5, this results in higher reflectivity and consequently lower output coupling at shorter wavelengths (see Supplementary Fig. S2). We measure the reflectivity to be ~ 80% at 1520 nm, decreasing to less than 50% at 1570 nm. The fiber-coupled off-chip output power is measured to be 0.06 mW at 1515 nm and increases to 1.78 mW at 1571 nm. The side-mode suppression ratio (SMSR) across the tuning range is plotted in Fig. 2(c), approaching ~ 70 dB at 1555 nm. The apparent decrease in SMSR at shorter wavelengths is attributed to reduced laser output power and approaching the sensitivity limit of the optical spectrum analyzer (see Supplementary Fig. S3).

### Frequency noise

**Fig. 3:**
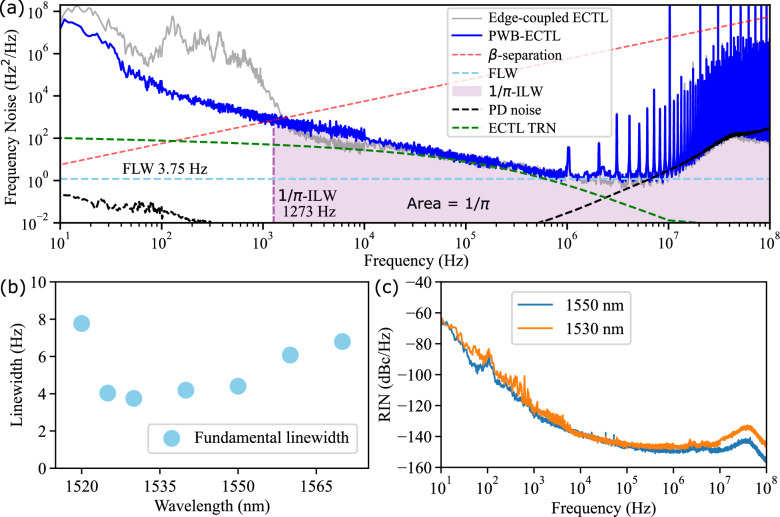
(**a**) Frequency noise (FN) of the photonic wirebond external cavity tunable laser (PWB-ECTL) is measured using a 1.026 MHz unbalanced fiber MZI as an optical frequency discriminator (OFD) and reading out the self-delayed homodyne signal on a high-speed photodetector (PD). The FN of the PWB-ECTL operating at 1530 nm (blue-solid) falls along the calculated estimate of the thermorefractive noise floor (TRN) of the intracavity rings (green-dashed line) before flattening out between 1 – 10 MHz frequency offset where we measure a fundamental linewidth (FLW) of 3.75 Hz, indicated by the blue-dashed line. The 1/π integral linewidth (ILW) is calculated using the reverse integration method and is indicated by the purple shading under the FN curve and stops at 1.27 kHz. The FN of an edge-coupled ECTL is shown as a comparison (gray-solid) and shows three orders of magnitude higher FN at ~ 100 Hz frequency offset as compared to the PWB integrated device. The beta-separation linewidth (red-dashed) of the edge-coupled and PWB-integrated ECTL is measured to be 151.5 kHz and 38.7 kHz, respectively. The spikes in the FN curves correspond to the free spectral range (FSR) of the OFD fiber-MZI, and the black-dashed curve indicates the noise floor of the photodetector (PD). (**b**) FLW of the PWB-ECTL measured across the tuning range. (**c**) Relative intensity noise (RIN) of the PWB-ECTL measured at two representative wavelengths. At a 1 MHz frequency offset, the RIN is -150 dBc/Hz at 1550 nm and -148 dBc/Hz at 1530 nm.

The frequency noise spectrum (FN) of the PWB-ECTL operating at 1530 nm is plotted in Fig. 3(a). We measure the FN of the laser using a 1 MHz unbalanced fiber-Mach Zehnder Interferometer (MZI) as an optical frequency discriminator (OFD) and measure the self-delayed homodyne signal on a high-speed photodetector. The sharp peaks at harmonics of 1 MHz are a measurement artifact from the fiber-MZI^2^. The fundamental linewidth (FLW) of the laser – corresponding to the white frequency noise floor, identified between 1–10 MHz frequency offset from the carrier – is 3.75 Hz, or 1.19 Hz^2^/Hz, and indicated by the blue-dashed horizontal line in Fig. 3(a). Between 30 kHz and 1 MHz frequency offset the FN follows the calculated thermorefractive noise floor (TRN) of the Vernier rings indicated by the dark green dashed curve. The integral linewidth (ILW) is 1.27 kHz, calculated using the 1/π reverse integration method^10^, and is illustrated by the purple shading under the FN curve. For comparison, the FN of a stage edge-coupled ECTL^2^ is shown in gray, where at low frequency offsets the FN exceeds that of the PWB-integrated device by as much as three orders of magnitude. This difference in FN is consistent with repeated measurements across the tuning range, see Supplementary Fig. S4. We measure the beta-separation linewidth of the edge-coupled ECTL to be 151.5 kHz as compared to 38.7 kHz for the PWB-ECTL. The FLW of the PWB-ECTL measured across the tuning range of the laser, shown in Fig. 3(b), ranges from 3.75–7.77 Hz. The FLW decreases slightly at lower wavelengths due to weaker ring-bus coupling in the intracavity rings, resulting in a longer effective laser cavity length. This is offset below 1520 nm by an increase in the photodetector noise due to the lower laser output power at shorter wavelengths. We also measure the laser relative intensity noise (RIN) at two representative wavelengths, plotted in Fig. 3(c).

## Discussion

Photonic wirebonding is a compelling approach to hybrid laser integration offering flexibility in connecting devices fabricated on different material platforms. Here, we show that PWB is a high-performance solution that maintains low fundamental noise and wide tunability while mitigating the low-to mid-frequency noise for a packaged InP/Si_3_N_4_ widely tunable, narrow linewidth ECTL laser design. The PWB-packaged laser exhibits a 3.75–7.77 Hz fundamental linewidth measured across a 60 nm tuning range and shows suppression of technical noise in the low frequency range below ~ 1 kHz compared to a stage edge-coupled version, a consequence of the improved optomechanical coupling stability offered by PWB, which has recently been demonstrated to survive the shock, vibration, radiation, and temperature cycling conditions of spaceflight^11^. The flexibility of the PWB permits modular design, removes the need for bespoke layouts for individual components, and accommodates a wide range of ECTL operating wavelengths. The modular approach to PICs is further enabled by PWB as the tight alignment tolerances between waveguides on separate chips are relaxed, accommodating height and lateral offsets exceeding 100 µm with acceptable loss levels.

The output power of the PWB-ECTL is comparable to that of edge-coupled implementations and can be improved through several pathways. Optimization of the PWB coupling loss – particularly through refined localization of the waveguide layer depth during writing and waveguide tapers more compatible with the PWB constraints – represents the most direct route. Complementary improvements include tuning the Sagnac loop mirror reflectivity to balance intracavity power against output power, and incorporating additional semiconductor gain blocks. Photonic wirebonding is particularly well suited to multi-gain architectures, as it greatly simplifies the challenge of connecting multiple RSOA or laser chips to a single silicon nitride external cavity. Dual-gain hybrid-integrated lasers have already yielded output powers exceeding 100 mW^12^, and have been demonstrated successfully with PWB integration^8^.

Although this work demonstrates operation at ~ 1550 nm, the cured polymer has a transparency window from 780 nm to 1.6 µm, and the technique can be applied across this range to accommodate a wide variety of high-performance ECTL designs for quantum^13,14^ and other precision applications. Towards shorter wavelengths, the minimum writable diameter (~ 1.6 µm) results in a multimode bond, though losses below 2 dB remain achievable at 780 nm. This work establishes a clear path toward narrow linewidth, widely tunable, packaged integrated lasers spanning the visible to short-wave IR for portable, high-precision and quantum experiments.

## Supplementary Information

Supplementary Information.

## Data Availability

Data underlying the results presented in this paper are not publicly available at this time but may be obtained from the authors upon reasonable request.
