# Supplementary material for: Narrow-linewidth photonic wirebonded silicon nitride external cavity tunable laser
Source: Sci Rep. 2026 May 1;16:20245. doi: 10.1038/s41598-026-50776-9 (PMC13323695; doi:10.1038/s41598-026-50776-9)
Supplement: Supplementary file 1 — Supplementary Information. [file 41598_2026_50776_MOESM1_ESM.docx]

**Narrow-linewidth photonic wirebonded silicon nitride external cavity tunable laser**

**David A. S. Heim^1^, Gar-Wing Truong^2^, Debapam Bose^1^, Eduardo Diaz^2^, Juan Ramirez^2^, Jes Sherman^2^, Gordon Morrison^2^, And Daniel J. Blumenthal^1,^***

*^1^Department of Electrical and Computer Engineering, University of California Santa Barbara, Santa Barbara, California 93106, USA*

*^2^Freedom Photonics, 41 Aero Camino, Santa Barbara, CA 93117, USA*

[*^*^danb@ucsb.edu*](mailto:*danb@ucsb.edu)

**Supplementary Information**

1. **Introduction**

This supplementary information provides additional information on the design and performance of the narrow linewidth photonic wirebonded (PWB) external cavity tunable laser (ECTL): including a table comparing this work to prior hybrid-integrated lasers, details on the PWB-RSOA coupling loss, the wavelength dependence of the Sagnac loop mirror reflectivity, the laser output power, and frequency noise as compared with a stage edge-coupled device.

1. **Comparison with prior hybrid-integrated lasers**

We compare characteristics and performance of the PWB-ECTL presented in this work with other hybrid-integrated lasers, including side mode suppression ratio (SMSR), output power, tuning, frequency noise (FN) at 1000 Hz and 100 Hz frequency offset, integral linewidth, and fundamental linewidth.

**Table S1. Comparison of hybrid-integrated lasers**

| Platform | Laser type | λ (nm) | FLW (Hz) | ILW (Hz) 1/π | FN @100 Hz offset (Hz^2^/Hz) | FN @1 kHz offset (Hz^2^/Hz) | Tuning (nm) | Output power (mW) | SMSR (dB) |
| --- | --- | --- | --- | --- | --- | --- | --- | --- | --- |
| Si_3_N_4_ [1] | SIL | 1550 | 0.04 | 236* | 192 | 30.5 | 0.8 | 0.3 | 60 |
| Si_3_N_4_ [2] | SIL | 1550 | 3.8 | 4,715* | … | 3.58E+5 | … | 10.5 | 65 |
| Si_3_N_4_ [3] | SIL | 1550 | 3 | 1,560* | 6.21E+6 | 1.11E+3 | … | … | 54 |
| Si_3_N_4_ [4] | SIL | 780 | 0.74 | 864 | 3.78E+4 | 561 | 2 | 2 | 36 |
| Si_3_N_4_ [5] | SIL | 785 | 700 | 50,173* | 3.78E+5 | 1.31E+6 | 12 | 10 | 37 |
| Si_3_N_4_ [6] | EDBR | 1550 | 320 | 47,466* | … | 1.95E+8 | … | 24 | 55 |
| Si [7] | ECTL | 1550 | 220 | 33,246* | … | … | 110 | 3 | 50 |
| Si [8] | ECTL | 1550 | 95 | 9,237* | 1.25E+5 | 2.37E+4 | 120 | 1.5 | 60 |
| Si_3_N_4_ [9] | ECTL | 1550 | 40 | 87,844** | … | … | 70 | 23 | 60 |
| Si_3_N_4_ [10] | ECTL | 1550 | 2,200 | 57,526* | … | 2.79E+8 | 120 | 24 | 63 |
| Si_3_N_4_ [11] | ECTL | 1550 | 750 - 4,000 | 31,614* | 6.99E+9 | 2.35E+7 | 172 | 26 | 68 |
| Si_3_N_4_ [12] | ECTL | 852 | 65 | 6,770* | 2.34E+8 | 6.57E+6 | 15 | 25 | 50 |
| Si_3_N_4_ [13] | ECTL | 1550 | 6 - 9.8*** | 2,350 | … | 1.46E+4 | 40 | 4.8 | 64 |
| Si_3_N_4_ [14] | ECTL | 1550 | 3 - 7*** | 1750 | 6.71E+6 | 4.22E+4 | 60 | 4.4 | 65 |
| Si_3_N_4_ [15] | PWB-ECTL | 1550 | 105000 | 141E+6* | … | … | 50 | 2.0 | 40 |
| TFLN [16] | PWB-ECTL | 1550 | 550 | 27,376* | 3.8E+5 | 9.39E+4 | 44 | 76.2**** | 61 |
| Si_3_N_4_ [17] | PWB-ECTL | 1550 | 979 | 39,245* | … | 2.36E+6 | 90 | 15.8**** | 59 |
| **Si_3_N_4_^†^** | **PWB-ECTL** | **1550** | **3.7 - 7.8***** | **1,256** | **2.51E+4** | **708** | **60** | **1.7** | **69.5** |

^†^ This work

* Not reported in manuscript: calculated from published frequency noise (FN) data

** ILW limited by available FN data

*** Measured across tuning range

**** On-chip power

1. **PWB losses**

The PWB optically connects the reflective semiconductor optical amplifier (RSOA) and the low loss silicon nitride (Si_3_N_4_) extended circuit while also enabling mode-matching between the two components of the laser cavity. Optical losses can occur at the RSOA-PWB interface, in the PWB -- due to mode tapering, absorption, bending or scattering -- and at the PWB-ECTL interface.

The coupling loss of the PWB in the final ECTL device was not characterized directly, however, the coupling loss in a test assembly comprising a similar RSOA and a Si_3_N_4_ waveguide with a similar input taper, was measured directly, and determined to be approximately 7 dB. Mode matching simulations of the RSOA-PWB and PWB-ECTL interface suggest achievable coupling losses of 0.6 dB and 0.7 dB, respectively, giving a total ideal mode-matching-induced loss of 1.3 dB. This is not inclusive of propagation or bending losses through the PWB.

The significant difference between the simulated and measured values is can be attributed to several factors: one, the non-optimal waveguide geometry. As noted in the text, the 18 μm-wide ECTL input taper was designed for free-space edge coupling to the RSOA and creates a much larger mode than is typical for photonic wirebonding. This necessitates an aggressive taper in the mode conversion section of the PWB since the entire bond length (which includes two tapers) is limited to the ~320 μm dimension of the writeable volume. Another factor is the bond placement. The total SiO_2_ cladding thickness in the a Si_3_N_4_ chip is 20 μm, and the waveguide core is only 80 nm thick, reducing the contrast available to the PWB tool’s confocal imager for locating the nitride/oxide interfaces. This led to a positional uncertainty of ~1 μm in the PWB placement (as determined by independent imaging modes of the PWB tool). Simulations of the mode-matching loss at the PWB-ECTL interface (shown below) indicate that a ~1.5 μm vertical displacement from the optimal position results in approximately 7 dB of excess loss – noting that this estimate accounts for mode-mismatch only and does not include taper, propagation, or bend losses.


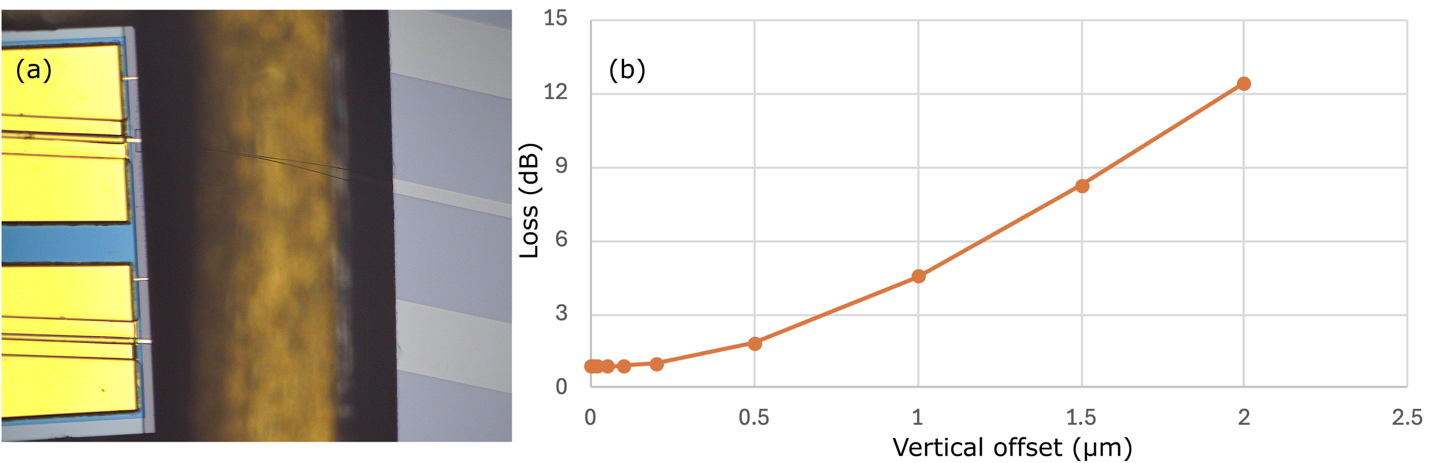


**Supplemental Fig. S1. Photonic wirebond coupling.** **a** Microscope image of the reflective semiconductor optical amplifier (RSOA) connected via photonic wirebond (PWB) to a silicon nitride waveguide photonic integrated circuit (PIC). **b** Simulation of the mode-mismatch induced loss at the PWB-PIC interface due to non-ideal vertical displacement from the optimal position.

1. **PWB-ECTL mirror reflectivity and output power**

The silicon nitride Sagnac loop mirror is a broadband reflector that consists of an evanescent directional coupler and a loop waveguide, and serves as the front mirror of the ECTL. The mirror reflectivity is a key design parameter, where high mirror reflectivity is beneficial for lowering the lasing threshold and supporting narrow fundamental linewidths, but does so at the expense of output coupling efficiency. For an idealized Sagnac mirror the reflectivity approaches unity for a directional coupler with power coupling coefficient $\kappa^{2}$ = 0.5. For our waveguide dimension (80 nm thick and 2.8 μm wide) with a gap of 2.5 μm and a coupling length of ~575 μm the directional coupler has a simulated $\kappa^{2}$ of 0.7 at 1515 nm and 0.9 at 1575 nm (see Supplemental Fig. S2). The mirror reflectivity, for the operating wavelengths of our ECTL, therefore increases at shorter wavelengths ($\kappa^{2}$ gets closer to 0.5) from less than 50% at 1570 nm to ~ 80% at 1520 nm.


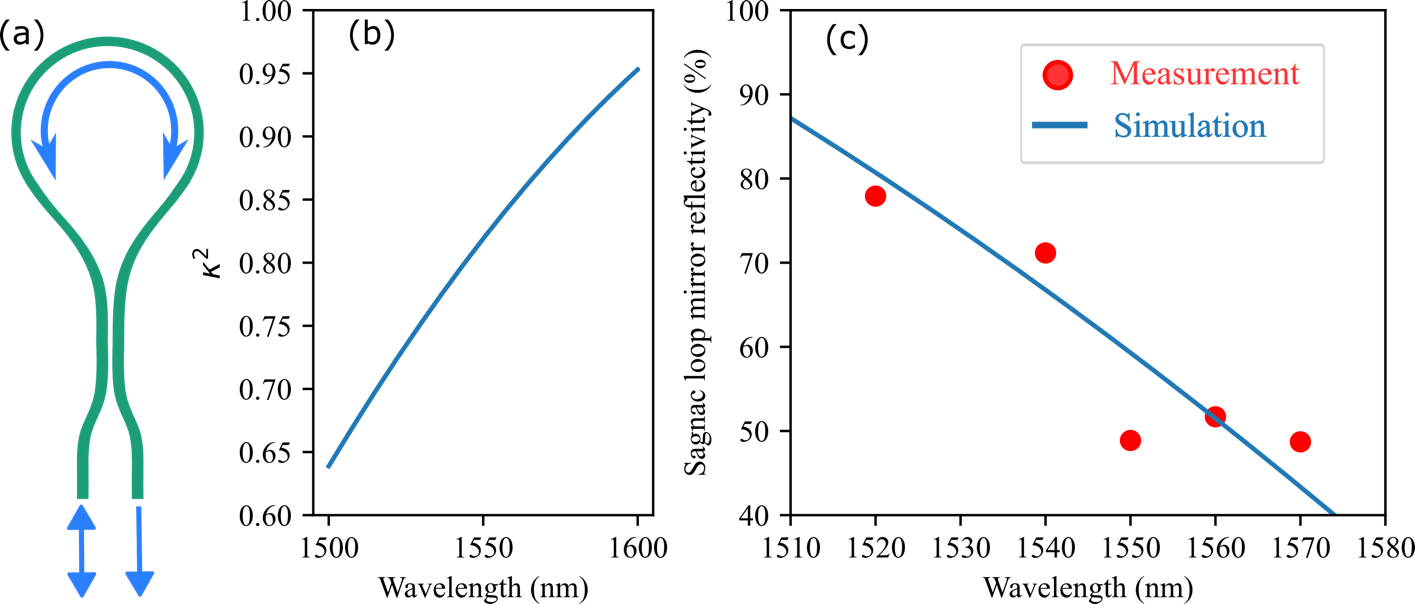


**Supplemental Fig. S2. Sagnac loop mirror.** **a** An illustration of the silicon nitride waveguide Sagnac loop mirror that serves as the front mirror of the external cavity tunable laser. **b** Simulation of the power coupling coefficient ($\kappa^{2}$) of the evanescent directional coupler in the Sagnac loop mirror. **c** Measurement and simulation of the Sagnac loop mirror reflectivity across operating wavelength range of the PWB-ECTL.

The output power of the PWB-ECTL is also wavelength dependent, due in part to the changing reflectivity of the loop mirror as demonstrated above, as well as changes in the ring resonator ring-bus coupling, and the gain spectrum of the RSOA. We measure the fiber-coupled output power of the PWB-ECTL with an optical power meter, and also measure the side mode suppression ratio (SMSR) on an optical spectrum analyzer (OSA), both are plotted in Supplemental Fig. S3.


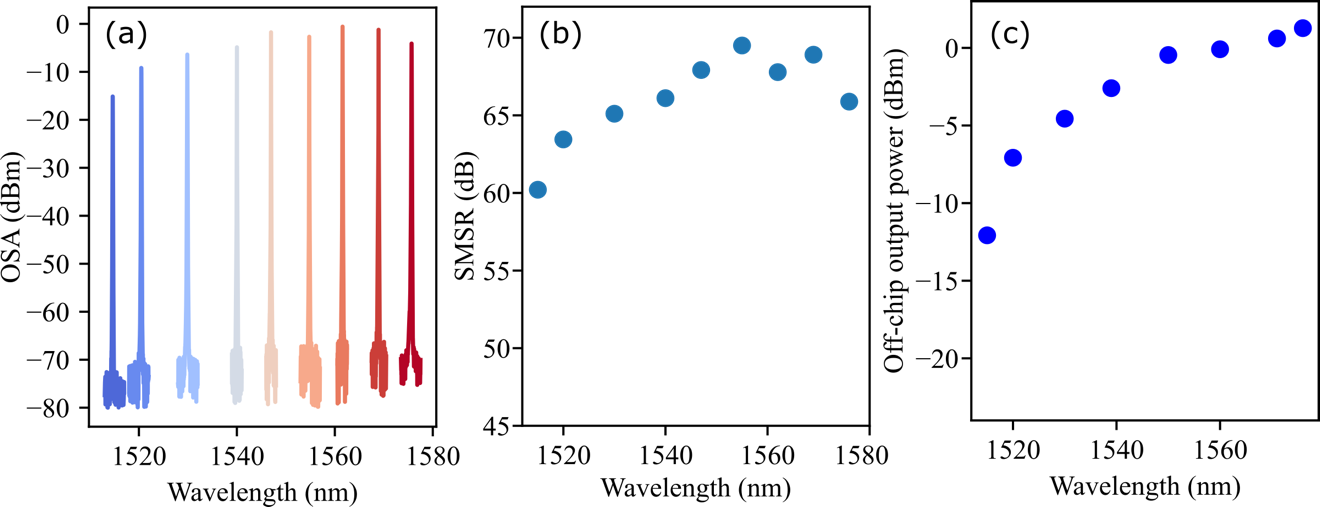


**Supplemental Fig. S3. PWB-ECTL sidemode suppression and output power. a** Laser spectra measured on an optical spectrum analyzer (OSA) across the PWB-ECTL tuning range. **b** Side mode suppression ratio (SMSR) calculated from the OSA spectra. **c** Off-chip fiber-coupled output power measured with an optical power meter.

1. **Frequency noise**

We compare the laser frequency noise of the PWB-ECTL with that of a stage, edge-coupled ECTL. Both the edge-coupled and PWB laser devices are from the same fabrication run, were tested on the same optical table, using the same frequency noise measurement setup (a 1.03 MHz fiber MZI used as an optical frequency discriminator), and operating at the same wavelengths. Although there is always some instance-to-instance variation in the measured frequency noise spectra, we highlight the consistency of these results from measurement to measurement, and across a wide wavelength range, by plotting below the frequency noise (FN) measurements of the edge-coupled and PWB ECTL devices at four different wavelengths (Supplemental Fig. S4). The traces at 1530 nm for each device are the two presented in the manuscript. The difference in FN at low frequency offsets between the edge-coupled and PWB device is as much as 1000x, much greater than the instance-to-instance variation in FN, and therefore we attribute the improvement to the superior coupling stability of the PWB as compared to the stage, edge-coupled case. This appears to be a plausible explanation as coupling drift and noise often appear in the low frequency band below 1 kHz offset, driven by slow thermal changes, and acoustic and seismic vibrations. Prior work [18] has directly investigated the robustness of the PWB approach as it relates to surviving the shock, vibration, radiation, and temperature cycling conditions of spaceflight.


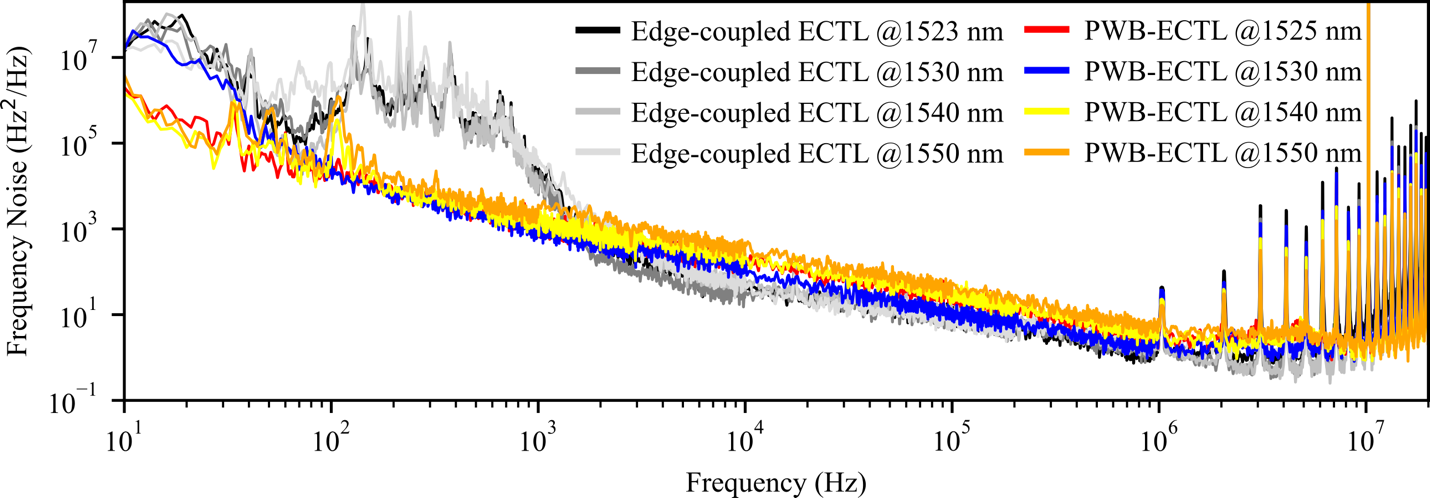


**Supplemental Fig. S4. Frequency noise.** Frequency noise (FN) measurements of the PWB-ECTL as compared with those of a similar stage, edge-coupled ECTL, highlighting the superior performance of the PWB-integrated device at low frequency offsets. At key frequency offsets of 100 Hz and 1000 Hz the FN of the PWB-ECTL is 2.51x10^4^ and 708 Hz^2^/Hz, respectively, as compared to 6.71x10^6^ and 4.22x10^4^ Hz^2^/Hz, respectively, for the stage, edge-coupled ECTL.

**References**

1. B. Li, W. Jin, L. Wu, L. Chang, H. Wang, B. Shen, Z. Yuan, A. Feshali, M. Paniccia, K. J. Vahala, and J. E. Bowers, "Reaching fiber-laser coherence in integrated photonics," Opt. Lett., OL **46**(20), 5201–5204 (2021).

2. A. Siddharth, A. Attanasio, S. Bianconi, G. Lihachev, J. Zhang, Z. Qiu, A. Bancora, S. Kenning, R. N. Wang, A. S. Voloshin, S. A. Bhave, J. Riemensberger, and T. J. Kippenberg, "Piezoelectrically tunable, narrow linewidth photonic integrated extended-DBR lasers," Optica, OPTICA **11**(8), 1062–1069 (2024).

3. C. Xiang, J. Guo, W. Jin, L. Wu, J. Peters, W. Xie, L. Chang, B. Shen, H. Wang, Q.-F. Yang, D. Kinghorn, M. Paniccia, K. J. Vahala, P. A. Morton, and J. E. Bowers, "High-performance lasers for fully integrated silicon nitride photonics," Nat Commun **12**(1), 6650 (2021).

4. A. Isichenko, A. S. Hunter, D. Bose, N. Chauhan, M. Song, K. Liu, M. W. Harrington, and D. J. Blumenthal, "Sub-Hz fundamental, sub-kHz integral linewidth self-injection locked 780 nm hybrid integrated laser," Sci Rep **14**(1), 27015 (2024).

5. M. Corato-Zanarella, A. Gil-Molina, X. Ji, M. C. Shin, A. Mohanty, and M. Lipson, "Widely tunable and narrow-linewidth chip-scale lasers from near-ultraviolet to near-infrared wavelengths," Nat. Photon. **17**(2), 157–164 (2023).

6. C. Xiang, P. A. Morton, and J. E. Bowers, "Ultra-narrow linewidth laser based on a semiconductor gain chip and extended Si_3_N_4_ Bragg grating," Opt. Lett., OL **44**(15), 3825–3828 (2019).

7. M. A. Tran, D. Huang, J. Guo, T. Komljenovic, P. A. Morton, and J. E. Bowers, "Ring-Resonator Based Widely-Tunable Narrow-Linewidth Si/InP Integrated Lasers," IEEE Journal of Selected Topics in Quantum Electronics **26**(2), 1–14 (2020).

8. P. A. Morton, C. Xiang, J. B. Khurgin, C. D. Morton, M. Tran, J. Peters, J. Guo, M. J. Morton, and J. E. Bowers, "Integrated Coherent Tunable Laser (ICTL) With Ultra-Wideband Wavelength Tuning and Sub-100 Hz Lorentzian Linewidth," Journal of Lightwave Technology **40**(6), 1802–1809 (2022).

9. Y. Fan, A. van Rees, P. J. M. van der Slot, J. Mak, R. M. Oldenbeuving, M. Hoekman, D. Geskus, C. G. H. Roeloffzen, and K.-J. Boller, "Hybrid integrated InP-Si_3_N_4_ diode laser with a 40-Hz intrinsic linewidth," Opt. Express, OE **28**(15), 21713–21728 (2020).

10. A. van Rees, Y. Fan, D. Geskus, E. J. Klein, R. M. Oldenbeuving, P. J. M. van der Slot, and K.-J. Boller, "Ring resonator enhanced mode-hop-free wavelength tuning of an integrated extended-cavity laser," Opt. Express, OE **28**(4), 5669–5683 (2020).

11. Y. Guo, X. Li, M. Jin, L. Lu, J. Xie, J. Chen, and L. Zhou, "Hybrid integrated external cavity laser with a 172-nm tuning range," APL Photonics **7**(6), 066101 (2022).

12. H. Nejadriahi, E. Kittlaus, D. Bose, N. Chauhan, J. Wang, M. Fradet, M. Bagheri, A. Isichenko, D. Heim, S. Forouhar, and D. J. Blumenthal, "Sub-100 Hz intrinsic linewidth 852 nm silicon nitride external cavity laser," Opt. Lett., OL **49**(24), 7254–7257 (2024).

13. Y. Wu, S. Shao, L. Tang, S. Yang, H. Chen, and M. Chen, "Hybrid integrated tunable external cavity laser with sub-10 Hz intrinsic linewidth," APL Photonics **9**(2), 021302 (2024).

14. D. A. S. Heim, D. Bose, K. Liu, A. Isichenko, and D. J. Blumenthal, "Hybrid integrated ultra-low linewidth coil stabilized isolator-free widely tunable external cavity laser," Nat Commun **16**(1), 5944 (2025).

15. Y. Xu, P. Maier, M. Blaicher, P.-I. Dietrich, P. Marin-Palomo, W. Hartmann, Y. Bao, H. Peng, M. R. Billah, S. Singer, U. Troppenz, M. Moehrle, S. Randel, W. Freude, and C. Koos, "Hybrid external-cavity lasers (ECL) using photonic wire bonds as coupling elements," Sci Rep **11**(1), 16426 (2021).

16. C. A. A. Franken, R. Cheng, K. Powell, G. Kyriazidis, V. Rosborough, J. Musolf, M. Shah, D. R. Barton III, G. Hills, L. Johansson, K.-J. Boller, and M. Lončar, "High-power and narrow-linewidth laser on thin-film lithium niobate enabled by photonic wire bonding," APL Photonics **10**(2), 026107 (2025).

17. P. Maier, Y. Chen, Y. Xu, Y. Bao, M. Blaicher, D. Geskus, R. Dekker, J. Liu, P.-I. Dietrich, H. Peng, S. Randel, W. Freude, T. J. Kippenberg, and C. Koos, "Sub-kHz-Linewidth External-Cavity Laser (ECL) With Si_3_N_4_ Resonator Used as a Tunable Pump for a Kerr Frequency Comb," J. Lightwave Technol., JLT **41**(11), 3479–3490 (2023).

18. J. Sherman, V. Rosborough, R. Gans, J. Ramirez, D. Kebort, G. Sitwell, J. Musolf, H. Garrett, T. Liu, C. McEwen, T. Cooper, A. Nehrir, G. Morrison, L. Johansson, and M. Mashanovitch, "Enabling space-qualified opto-electronic systems through photonic wirebonding," in *Sensors and Systems for Space Applications XVII* (SPIE, 2024), **13062**, pp. 34–41.
